# Echocardiography as a Screening Test for Myocardial Scarring in Children with Hypertrophic Cardiomyopathy

**DOI:** 10.1155/2016/1980636

**Published:** 2016-11-15

**Authors:** Gregory Compton, Lynne Nield, Andreea Dragulescu, Lee Benson, Lars Grosse-Wortmann

**Affiliations:** ^1^Department of Diagnostic Imaging, The Hospital for Sick Children, University of Toronto, 555 University Avenue, Toronto, ON, Canada; ^2^Department of Pediatrics, Division of Cardiology, The Hospital for Sick Children, University of Toronto, 555 University Avenue, Toronto, ON, Canada

## Abstract

*Introduction*. Hypertrophic cardiomyopathy (HCM) is burdened with morbidity and mortality including tachyarrhythmias and sudden cardiac death. These complications are attributed in part to the formation of proarrhythmic scars in the myocardium. The presence of extensive LGE is a risk factor for adverse outcomes in HCM. Late gadolinium enhancement (LGE) cardiac magnetic resonance imaging (cMRI) is the standard for the noninvasive evaluation of myocardial scars. However, echocardiography represents an attractive screening tool for myocardial scarring. The aim of this study was to compare the suitability of echocardiography to detect myocardial scars to the standard of cMRI-LGE.* Methods*. The cMRI studies and echocardiograms from 56 consecutive children with HCM were independently evaluated for the presence of cMRI-LGE and echocardiographic evidence of scarring by expert readers.* Results*. Echocardiography had a high sensitivity (93%) and negative predictive value (94%) in comparison to LGE. The false positive rate was high, leading to a low specificity (37%) and a low positive predictive value (35%).* Conclusions*. Given the poor specificity and positive predictive value, echocardiography is not a suitable screening test for the presence of myocardial scarring in children with HCM. However, children without echocardiographic evidence of myocardial scarring may not need to undergo cardiac magnetic resonance imaging to “rule in” LGE.

## 1. Introduction

Hypertrophic cardiomyopathy (HCM) is the most common cardiomyopathy, occurring in approximately 1 in 500 individuals [1–5] and affecting both sexes equally with no ethnic preponderance [5]. HCM is the most common cause of sudden cardiac death in young adults [1–8]. Myocardial scarring has been identified as an important risk factor for ventricular arrhythmias [6] and sudden cardiac death in those with HCM [9]. Cardiac magnetic resonance imaging (cMRI), using late gadolinium enhancement (LGE), is the gold standard noninvasive method for the detection of focal myocardial fibrosis [10]. The presence of LGE is associated with adverse clinical events [11, 12] in both adult and pediatric patients [2, 13] including progressive ventricular dilation [14], ventricular tachyarrhythmias [6], and sudden cardiac death [15].

Clinical practice suggests that scars in the myocardium may also be suspected based on cardiac ultrasound: fibrotic myocardium appears hyperechoic due to increased reflectivity of the ultrasound waves, in comparison to nonfibrotic myocardium. This difference is thought to be due to the concentration of collagen within fibrotic myocardium causing increased reflectivity [16, 17]. Ultrasound has been utilized for the qualitative and quantitative assessment of hepatic fibrosis [18]. However, to date, no systematic analysis of its usefulness in the detection of myocardial scarring in comparison with cMRI-LGE has been undertaken. In this study, we sought to compare the detection of fibrosis by echocardiography to LGE-cMRI in a pediatric population with HCM.

## 2. Materials and Methods

Following approval by the institutional research ethics board, imaging studies from 56 consecutive children with HCM who had undergone both cMRI and echocardiography were retrospectively reviewed while serial studies in the same patient were excluded from analysis. Patient demographic data can be found in Table 1. Our cohort included patients with suspected or confirmed sarcomeric HCM. Patients with syndromic, neuromuscular, or storage diseases were excluded from the study. All CMR scans were performed on a 1.5 T scanner (“Avanto,” Siemens Medical Systems, Erlangen, Germany), following a uniform protocol which included sequences for analysis of ventricular volumetry and LGE. In brief, for LGE, stacks of 5 images were obtained in the short axis orientation, covering the LV from base to apex using a segmented inversion recovery technique with phase sensitive inversion recovery reconstruction. The slice thickness was 6 mm and the in-plane resolution was 1.4 mm × 1.4 mm. Suitable inversion times to null the myocardial signal were derived from T1 scout sequences and the trigger delay was set to image in diastole. A more thorough description of this standard techniques can be found elsewhere [19]. Images were viewed on a Centricity PACS (GE Healthcare, Barrington, IL, USA) workstation.

Echocardiographic studies were performed using a Vivid 7 or E9 unit (GE Medical Systems, Milwaukee, WI, USA) or a Philips IE33 unit (Philips, Best, Netherlands). Images were obtained in standard parasternal and apical views and evaluated on a Syngo V5.1 workstation (Siemens Medical Solutions).

**Figure 1:**
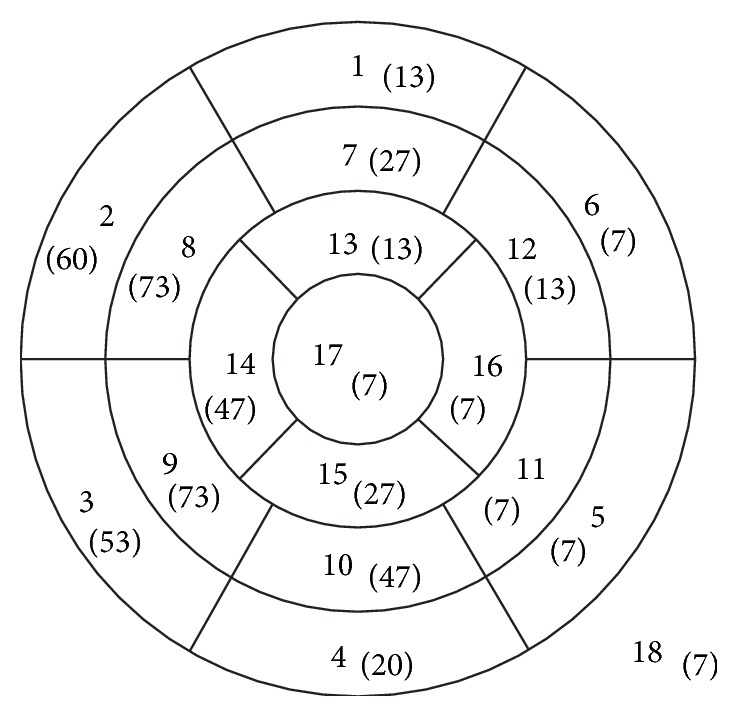
Schematic diagram of the AHA myocardial segments [20], with segment 18 representing the papillary muscles. The number in parentheses is the percentage of positive LGE for that segment in our patient population, with total patients who are LGE positive as the denominator.

The CMR examinations were interpreted by an experienced senior reader (LGW) using the American Heart Association 17 segment model [20] to describe the location of LGE. For the purpose of this study papillary muscles were labeled as segment “18” (Figure 1).

**Figure 2:**
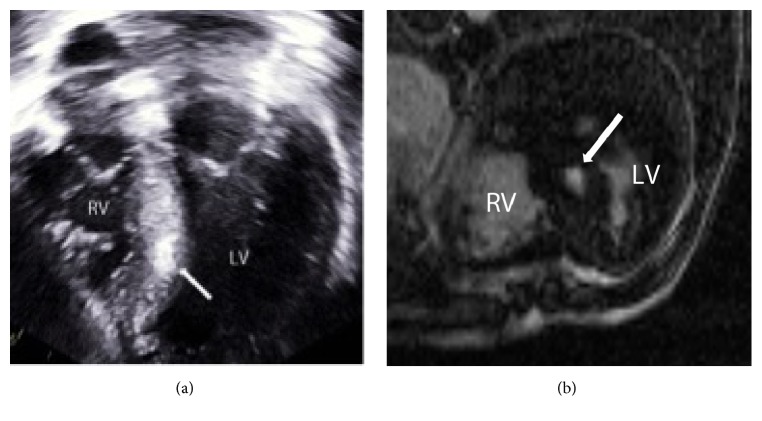
Sample images from echocardiography (a) and cMRI (b) performed on the same patient demonstrating positive findings (white arrows) of hyperechogenicity of the septal myocardium on echocardiography and LGE in the same location at cMRI (RV: right ventricle and LV: left ventricle).

For the assessment of LGE, the “magnitude images” of the acquisition were used. The reader was blinded to the clinical details and to the results of other testing. Fibrosis and myocardial scarring on ultrasound is identified as hyperechogenicity of the affected myocardium in comparison to background normal myocardium. The echocardiograms were interpreted by a senior echocardiography reader (LN) who was blinded to the results of the cMRI as well as the patients clinical details. This is similar to the appearance of hepatic fibrosis on ultrasound which is related to the severity of fibrosis [21]. An educational session was held prior to starting the analysis to demonstrate a case of positive LGE and the corresponding echocardiographic images (Figures 2(a) and 2(b)).

*Statistical Analysis*. Kappa correlation coefficients were calculated to assess the agreement between the 2 modalities both for all patients and secondarily for only those deemed positive by cMRI for myocardial fibrosis.

## 3. Results

The median time difference between the echocardiogram and cMRI was 30 days with a range of 0 days to 15 months. Late gadolinium enhancement was identified on cMRI in 15 (27%) of the 56 children. Based on a total of 1008 total segments assessed (including the papillary muscles as an additional 18th segment), LGE was seen in 76 segments or 7.5% of all segments analyzed. In the individual child the number of segments with LGE ranged from 1 to 15. In those with LGE, more than 2 segments were affected in 67% of the cohort. The myocardial segments most commonly affected were 8 and 9 (midanteroseptal and midinferoseptal), in 73% of the children, and segment 2 (basal-anteroseptal) was the next most commonly affected in 60% of the cohort. Segments 5, 6, 11, 16, and 17 (basal-inferolateral and anterolateral, midinferolateral, apical-lateral, and apical) and the papillary muscles were the least commonly affected.

The evaluation of the echocardiograms identified 40 of the 56 children (71%) as positive for the presence of myocardial fibrosis. One child who had LGE on cMRI was not identified by echocardiography. On the echocardiograms, myocardial fibrosis was most commonly suspected in segments 3 and 8 (basal-inferoseptal and midanteroseptal), both of these segments were positive in 87% of the ultrasound positive studies, with segment 14 (apical-septal) being the next most common at 73% of the studies. The least commonly affected segments in children deemed positive for myocardial fibrosis were segments 4 and 12 (basal-inferior and midanterolateral).

**Figure 3:**
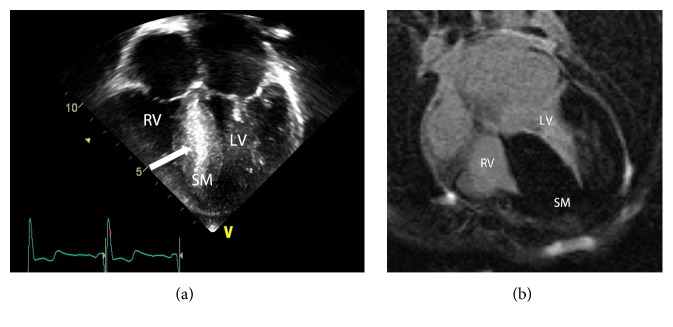
Sample images from echocardiography (a) and cMRI (b) performed on an 8-year-old male patient with HCM demonstrating a* false positive* result on echocardiography. (a) demonstrates increased echogenicity of the septal myocardium (white arrow) with no corresponding abnormality seen at cMRI; note the homogeneous signal of the septal myocardium. (RV: right ventricle, LV: left ventricle, and SM: septal myocardium).

**Figure 4:**
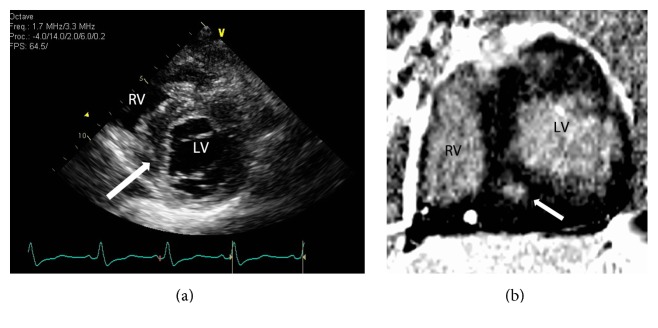
Sample short axis images from echocardiography (a) and cMRI (b) performed on a 12-year-old female patient with HCM demonstrating a* false negative* result on echocardiography. (a) demonstrates no corresponding focus of hyperechogenicity (white arrow) within the septal myocardium, which is apparent on cMRI (b) that confirms the presence of LGE within the same location indicating fibrosis. (RV: right ventricle and LV: left ventricle).

Kappa analysis comparing echocardiography and cMRI yielded an overall agreement (Po) of 0.53 for the presence or absence of myocardial fibrosis. The kappa value of 0.05 between the 2 modalities indicates poor agreement, similar to that of chance. When comparing the agreement for positive segments, kappa analysis yielded a value of 0.45 and moderate agreement with a Po of 0.73. The sensitivity of echocardiography for the detection of fibrosis, compared to cMRI detected LGE as the gold standard, was 93%, with a specificity of 37% (Table 2). The negative predictive value was 94% while the positive predictive value was 35%. The positive and negative likelihood ratios were 1.47 and 0.18. Examples of false positive and false negative results, that is, lack of positive LGE on cMRI identified as hyperechogenicity on echocardiogram and lack of identification of hyperechogenicity on echocardiography, can be seen in Figures 3 and 4, respectively.

## 4. Discussion

Late gadolinium enhancement on cMRI is the clinical standard for the identification, quantification, and assessment of the distribution of myocardial fibrosis, achieving excellent agreement with myocardial scarring on histology [22–24]. The hyperenhancement seen on LGE images is due to the accumulation of gadolinium contrast within the expanded interstitial (extracellular) space in the myocardial scar [14, 25]. In adults with HCM, extensive LGE appears to be a risk factor for arrhythmia and sudden cardiac death, independent of traditional risk factors [9]. Whether the presence of LGE holds prognostic value in children and adolescents is uncertain. Early data suggest that children with a higher extent of LGE have an increased level of adverse cardiac events in comparison to those children with a lesser degree of LGE [2] including sudden cardiac death, ventricular dilation, ventricular aneurysm formation, and ventricular tachyarrhythmias [11]. An LGE prevalence of 27% in our patient population is less than that previously reported in the literature for both pediatric and adult populations [2, 12, 13] where reports in children and adolescents have noted a prevalence of 57% and 73% [2, 13], respectively. The reason for the lower prevalence of LGE in the current cohort is uncertain. The discrepancy may in part be related to differences in patient selection (older patients in the Smith et al. study; higher prevalence of heart failure in the Chaowu et al. report).

The most commonly scarred myocardial segments by cMRI in our cohort were the basal-anteroseptal, midanteroseptal, and midinferoseptal segments. These segments include the right ventricular insertion points into the interventricular septum. These findings are similar to that reported in adult HCM patients [26], with these junctions as well as the septum itself previously identified as the areas most commonly involved in myocardial scarring in adult patients with HCM [25].

Despite its established role in the detection and quantification of myocardial scarring cMRI has limitations. It is time consuming and costly, requires intravenous access for the injection of contrast, and is not always readily available. Echocardiography on the other hand is not burdened by such limitations and at least in theory makes an attractive imaging modality as a screening test for fibrosis in HCM. The WHO criteria [27] for a good screening test are

- the condition screened for should be an important one,
- there should be an acceptable treatment for patients with the disease,
- the facilities for diagnosis and treatment should be available,
- there should be a recognized latent or early symptomatic stage,
- the screening should have high specificity and sensitivity,
- the test should be acceptable to the population,
- the cost, including diagnosis and subsequent treatment, should be economically balanced in relation to expenditure on medical care as a whole.

Echocardiographic screening for myocardial scarring fulfills many of these requirements: it is relatively inexpensive, readily available, and noninvasive. Myocardial fibrosis is present in a significant proportion (27% to 73%) of the pediatric population and there is data in adults and preliminary experience in children that LGE is associated with increased risk for adverse outcomes, including sudden cardiac death. Prior to life-threatening arrhythmias, many patients are asymptomatic; that is, there is a latent or early symptomatic stage as per the WHO criteria. Appropriate monitoring and the placement of an implantable defibrillator can abrogate the associated morbidity and mortality, due to sudden cardiac death and arrhythmias.

In this cohort, echocardiography identified myocardial scarring with a high sensitivity and negative predictive value when compared to the presence of LGE by cMRI. However, the specificity and positive predictive value of echocardiography in the detection of myocardial scarring were poor, as a result of a large number of false positives. As a consequence, the agreement between cMRI and echocardiography was not much better than chance and as such echocardiography screening cannot substitute for cMRI. Within the group of LGE-cMRI positive children there was a moderate level of agreement between echocardiography and cMRI with regard to the AHA segments that were either involved or spared. Another shortcoming of echocardiography is the fact that the extent of scarring, that is, the percentage of involved myocardium, cannot be quantified. Amount of LGE by cMRI, on the other hand, is a discriminator between increasing risk levels for adverse outcomes [9].

Despite these shortcomings echocardiography may be useful because of its role in the routine diagnostic work-up: virtually every patient with HCM undergoes serial echocardiography. As a consequence fibrosis screening by echocardiography, albeit far from perfect, does not incur an extra cost or burden for the patient. Furthermore, for a screening test, sensitivity and negative predictive value (which were high by echocardiography) are more important than sensitivity and positive predictive value. In their 2011 guidelines the American College of Cardiology Foundation and the American Heart Association recommend LGE-cMRI “when sudden cardiac death risk stratification is inconclusive after documentation of the conventional risk factors” as a Class IIb recommendation, level of evidence C [28]. One could argue that this applies to every patient in whom primary prevention of sudden cardiac death is the goal, which is true for children with HCM. Given echocardiography's excellent sensitivity and negative predictive value it may serve to reduce the number of patients who require a cMRI for the detection of LGE. Avoidance of contrast-enhanced cMRI is particularly important in those who have contraindications to the technique or in young children who would require sedation or general anesthesia to perform the cMRI study. In this regard, the number of children who had a cMRI to assess for myocardial scarring could have been reduced by 16 or 29% of the cohort studied here. Therefore, despite a high rate of false positives, screening the echocardiographic images for signs of fibrosis may be indicated, especially at institutions where cMRI is not a routine practice or not readily available [29].

## 5. Limitations

Several limitations of this study warrant discussion: firstly, the relatively low number of patients that had LGE on cMRI in the study population limits the statistical power. Secondly, the use of two different types of ultrasound machines used for the clinical tests in this retrospective study may have resulted in different imaging characteristics which may in turn have influenced the appearance and interpretation of myocardium as healthy or fibrosed. However, the cohort size was too small to assess this effect. Finally, while the median interval between echocardiography and cMRI was only 30 days the longest interval was slightly over a year. We elected to include those children with longer intermodality gaps if there was no change in clinical status, as myocardial scarring was thought not to change significantly over a span of months.

## 6. Conclusion

Echocardiography does not reliably identify myocardial fibrosis in children and adolescents with HCM as evidenced by cMRI-LGE. The poor performance of echocardiography is largely due to its high rate of false positives. Despite this shortcoming, and given the excellent negative predictive value and its role as the primary imaging modality in HCM, echocardiography may be useful in preselecting those who may benefit from cMRI imaging to either confirm or refute the finding of myocardial scarring.

## Figures and Tables

**Table 1:** Demographic information for study patients (median ± standard deviation).

Age (years)	12 ± 3
Male	46 (81%)
Duration between studies (days)	50 ± 74

**Table 2:** Comparison of CMR and echocardiography for the presence or absence of LGE/fibrosis.

	MRI	
	Positive	Negative
Echocardiography		
Positive	14	26
Negative	1	15
